# *Ishige okamurae* Extract Ameliorates the Hyperglycemia and Body Weight Gain of *db/db* Mice through Regulation of the PI3K/Akt Pathway and Thermogenic Factors by FGF21

**DOI:** 10.3390/md17070407

**Published:** 2019-07-09

**Authors:** Young-Jin Seo, Kippeum Lee, Sungwoo Chei, You-Jin Jeon, Boo-Yong Lee

**Affiliations:** Department of Food Science and Biotechnology, College of Life Science, CHA University, Seongnam, Kyeonggi 13488, Korea

**Keywords:** *Ishige okamurae*, insulin receptor substrate 1/phosphatidylinositol 3-kinase/Akt pathway, hyperglycemia, fibroblast growth factor 21, *db/db* mouse

## Abstract

Type 2 diabetes mellitus and related metabolic disorders, such as dyslipidemia, present increasing challenges to health worldwide, as a result of urbanization, the increasing prevalence of obesity, poor lifestyle, and other stress-related factors. *Ishige okamurae* extract (IOE) is known to be effective at lowering blood glucose and ameliorating metabolic disease. However, detailed mechanisms for these effects have yet to be elucidated. Here, we show that IOE ameliorates substrate (IRS)/ phosphatidylinositol 3-kinase (PI3K)/Akt pathway and increasing glucose transporter 4 (GLUT4) expression in skeletal muscle and white adipose tissue (WAT). We also demonstrate that IOE increases the expression of fibroblast growth factor (FGF)21, a regulator of glucose and energy metabolism in muscle and WAT. In addition, IOE administration increased peroxisome proliferator-activated receptor γ coactivator 1α expression, which regulates expression of the key thermogenic molecule uncoupling protein 1 in WAT. Thus, the effects of IOE to ameliorate hyperglycemia and adiposity may be mediated through FGF21 activating insulin signaling and increasing the expression of GLUT4 and pro-thermogenic factors.

## 1. Introduction

Type 2 diabetes is characterized by insulin resistance and hyperglycemia and is predisposed to by a number of factors, including obesity, inappropriate lifestyle, poor diet, and genetic factors [1,2]. Insulin resistance develops as a result of high circulating concentrations of fatty acids and their ectopic deposition in tissues, leading to lower glucose uptake into skeletal muscle [3,4]. The C57BlKsJ-*db/db* mouse (*db/db* mouse) is characterized by hyperglycemia and has been widely used as a model of type 2 diabetes and diabetic nephropathy. More specifically, the *db/db* mouse is a model of obesity-induced type 2 diabetes, because it demonstrates very high fat mass due to a mutation in its leptin receptor. Obesity is a significant risk factor for type 2 diabetes because it is associated with insulin resistance [5]. Therefore, weight loss can be effective at slowing or preventing the onset of type 2 diabetes.

Insulin is the key regulator of glucose metabolism in skeletal muscle and adipose tissue [6], and muscle is the major site of insulin-stimulated glucose disposal. However, white adipose tissue (WAT) has a crucial role in the regulation of insulin sensitivity through secretion of cytokines [7]. The binding of insulin to the insulin receptor initiates intracellular insulin signaling [8]. The receptor recruits and phosphorylates insulin receptor substrate 1 (IRS1), which binds phosphatidylinositol 3-kinase (PI3K), which in turn activates the downstream kinase Akt [9]. The PI3K/Akt pathway regulates glucose metabolism, glycogen synthesis, and cellular survival pathways [10]. In WAT and skeletal muscle, Akt stimulates translocation of glucose transporter 4 (GLUT4) to the cell surface, facilitating the entry of glucose into the cell. However, in diabetes, impaired translocation of GLUT4 to the cell membrane is associated with a reduction in glucose disposal into muscle and WAT [3]. Similarly, GLUT4 knockout impairs glucose tolerance and causes insulin resistance in these tissues [11,12].

Fibroblast growth factor 21 (FGF21) is mainly expressed in the liver, but also in other tissues, including WAT, skeletal muscle, and pancreas [13,14]. FGF21 has been suggested to be a potential therapy for obesity and type 2 diabetes, with several previous studies having shown that the induction of FGF21 expression leads to significant metabolic improvements, including reductions in fasting glucose, insulin, and triacylglycerol (TG) concentrations in high-fat diet (HFD)-fed and leptin-deficient obese mice [15,16,17]. The administration of FGF21 improves insulin sensitivity by upregulating AMP-activated protein kinase-Akt signaling in WAT and skeletal muscle [18,19]. Furthermore, FGF21 regulates the browning of adipose tissue and metabolism by regulating uncoupling protein (UCP)1 and peroxisome proliferator-activated receptor (PPAR) coactivator (PGC)1α expression, which are important for mitochondrial function and energy expenditure [20].

*Ishige okamurae*, a brown alga, has been shown to have several physiologic effects, including protection against oxidative stress and inflammation [21,22]. Administration of *Ishige okamurae* extract (IOE) has been shown to ameliorate hyperglycemia, but the mechanism of this effect on glucose homeostasis has not been determined. Here, we aimed to determine the effects of IOE on insulin signaling and GLUT4 expression in *db/db* mice and to compare them with those of metformin, the first-line drug for the treatment of type 2 diabetes [23]. We show that IOE may improve glucose homeostasis and hyperlipidemia by activating the IRS/PI3K/Akt pathway and increasing the expression of pro-thermogenic genes in muscle and WAT, as a result of greater FGF21 expression.

## 2. Results

### 2.1. Treatment with IOE Improves Glucose Tolerance and Insulin Sensitivity in Mice

**Figure 1:**
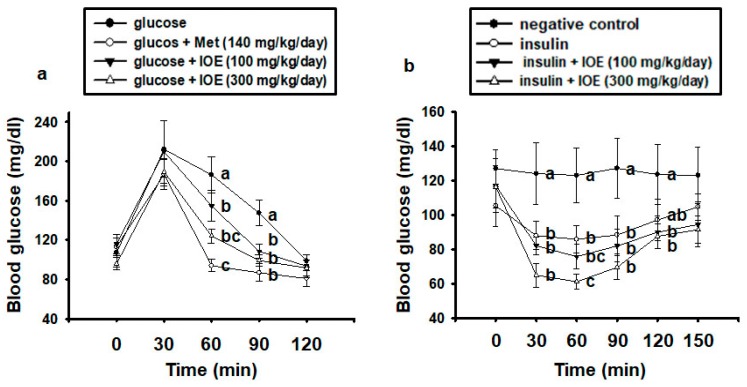
Treatment with *Ishige okamurae* extract (IOE) ameliorates glucose intolerance and insulin resistance in mice. Glucose tolerance and insulin sensitivity were assessed by (**A**) oral glucose tolerance test (OGTT) and (**B**) intraperitoneal insulin tolerance test (IPITT) in mice. All data are presented as mean ± SD (*n* = 6). Values with different letters are significantly different; *p* < 0.05 (a > b > c).

OGTT and IPITT are used to assess glucose tolerance and insulin sensitivity, and to identify hyperglycemia and type 2 diabetes [24,25]. We used OGTT to determine the effect of IOE treatment on glucose tolerance in mice. As shown in Figure 1A, every group demonstrated fasting glucose concentrations within the normal range at 0 min. Glucose administration caused a marked increase in blood glucose at 30 min, but at 60 and 90 min, the IOE-treated groups demonstrated a faster decline in blood glucose than the glucose-treated control group. Administration of 140 mg/kg metformin also significantly reduced blood glucose concentrations at 60 and 90 min compared with the control group. In addition, treatment with IOE resulted in glucose concentrations that were significantly lower than the concentrations in the control group at 60 and 90 min.

To determine whether IOE also improves insulin sensitivity in mice, we performed an IPITT. As shown in Figure 1B, the 300 mg/kg IOE group showed significantly greater reductions in blood glucose than did the insulin-treated control group at 60 min. These data suggest that IOE can ameliorate glucose intolerance and insulin resistance in *db/db* mice. 

### 2.2. IOE Administration Ameliorates the Hyperglycemia and Dyslipidemia of db/db Mice

**Figure 2:**
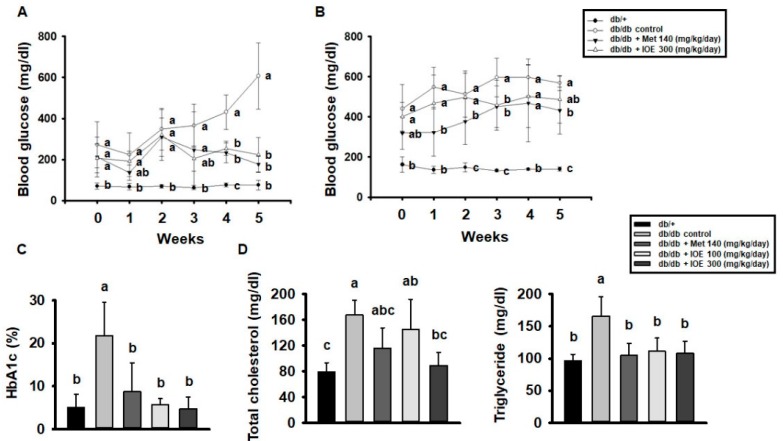
IOE administration ameliorates hyperglycemia and dyslipidemia in *db/db* mice. (**A**) Fasting blood glucose and (**B**) postprandial blood glucose after 5 weeks of IOE treatment in *db/db* mice. (**C**) HbA1c and (**D**) total cholesterol and triglyceride concentrations were measured using colorimetric kits. All data are expressed as mean ± SD (*n* = 6). Values with different letters are significantly different; *p* < 0.05 (a > b > c).

We next assessed the anti-hyperglycemic effect of long-term IOE administration in *db/db* mice. Over the 5 weeks period of the study, the fasting blood glucose concentrations of control *db/db* mice increased gradually (Figure 2A), but the concentrations were lower in the mice treated with IOE. In addition, the IOE groups showed significantly lower postprandial blood glucose levels than the control group (Figure 2B). As shown in Figure 2C,D, HbA1c, total cholesterol, and TG concentrations were higher in control *db/db* mice than in db/+ mice, but the IOE-treated groups had lower concentrations of all three than control *db/db* mice. Thus, 5 weeks of treatment with IOE ameliorates the hyperglycemia and dyslipidemia of *db/db* mice.

### 2.3. IOE Administration Increases GLUT4 Expression and Activates the Insulin Signaling Pathway in Skeletal Muscle

**Figure 3:**
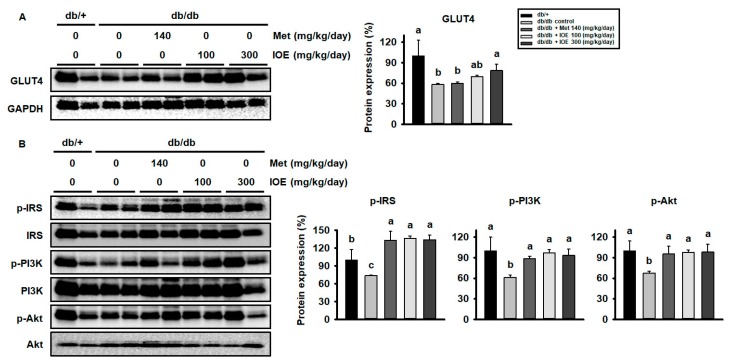
IOE administration increases GLUT4 expression and the phosphorylation of the PI3K signaling pathway intermediates in muscle. (**A**) GLUT4 expression was measured by western blotting of muscle lysates. (**B**) Phosphorylation of IRS, PI3K, and Akt in the muscle of *db/db* mice. All data are expressed as mean ± SD (*n* = 6). Values with different letters are significantly different; *p* < 0.05 (a > b > c).

To investigate the mechanism whereby IOE ameliorates hyperglycemia, we quantified the protein levels of signaling intermediates and GLUT4 in muscle by western blot analysis. As shown in Figure 3A, control *db/db* mice demonstrated lower GLUT4 protein expression than db/+ mice. However, GLUT4 expression was higher in the metformin and IOE-treated groups than in control mice. As shown in Figure 3B, *db/db* mice also demonstrated lower levels of phosphorylation of IRS1, PI3K, and Akt than db/+ mice, but IOE administration ameliorated these defects in phosphorylation. These data indicate that IOE may promote glucose uptake in the muscle of *db/db* mice by activating the insulin signaling pathway and increasing GLUT4 expression.

### 2.4. IOE Administration Also Increases GLUT4 expression and Activates the Insulin Signaling Pathway in WAT

**Figure 4:**
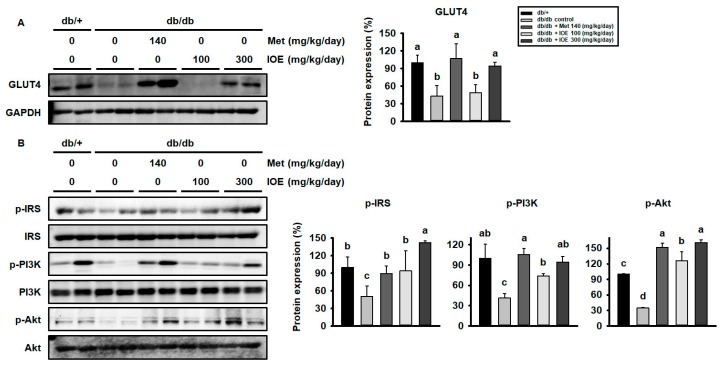
IOE administration upregulates GLUT4 expression and activation of the PI3K signaling pathway in white adipose tissue (WAT). (**A**) Western blotting for GLUT4 protein expression in the WAT of *db/db* mice. (**B**) Phosphorylation of IRS, PI3K, and Akt and their total protein expression in the WAT of *db/db* mice. All data are expressed as mean ± SD (*n* = 6). Values with different letters are significantly different; *p* < 0.05 (a > b > c > d).

We also determined whether the insulin signaling pathway is activated by IOE treatment in the WAT of diabetic mice. As shown in Figure 4A, *db/db* mice demonstrated lower levels of GLUT4 than db/+ mice, but the administration of either metformin or IOE was associated with higher GLUT4 expression. In addition, as shown in Figure 4B, control *db/db* mice demonstrated lower levels of phosphorylation of IRS1, PI3K, and Akt than db/+ mice, but consistent with the muscle data, IOE ameliorated these defects. These data indicate that IOE may enhance glucose uptake into the WAT of *db/db* mice by activating the insulin signaling pathway and increasing GLUT4 protein levels here.

### 2.5. IOE Ameliorates Obesity in db/db Mice

**Figure 5:**
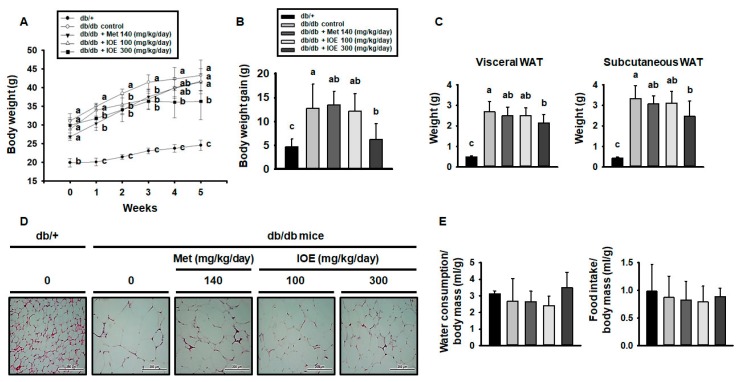
IOE treatment reduces adiposity in *db/db* mice. (**A**) Body mass change, (**B**) body mass gain, and (**C**) visceral and subcutaneous WAT masses were measured during 5 weeks of treatment with IOE, metformin, or vehicle. (**D**) WAT histology was evaluated after hematoxylin and eosin (H and E) staining. (**E**) Water and food intake per unit of body mass. All data are presented as mean ± SD (*n* = 6). Values with different letters are significantly different; *p* < 0.05 (a > b > c).

As shown in Figure 5A, *db/db* control mice gained body mass faster than db/+ mice, but the gain in body mass was prevented in the *db/db* mice by 1–5 weeks of IOE administration (Figure 5B). To identify the cause of the slower mass gain, the WAT depots of the mice were weighed, and the visceral and subcutaneous WAT masses were found to be higher in *db/db* mice than in db/+ mice (Figure 5C), but treatment with 300 mg/kg/day IOE substantially reduced this difference. Furthermore, as shown in Figure 5D, histologic assessment of WAT from db/+ mice showed that it consisted of cells containing relatively small fat droplets, whereas cells from *db/db* mice had larger fat droplets. However, in both the IOE and metformin-treated groups this difference was much smaller. There were no significant differences among the groups with regard to food or water consumption (Figure 5E). Thus, IOE administration ameliorates leptin receptor deficiency-induced obesity in *db/db* mice.

### 2.6. IOE Administration Increases the Expression of Proteins Involved in Adipocyte Browning, Perhaps by Upregulating FGF21 Secretion

**Figure 6:**
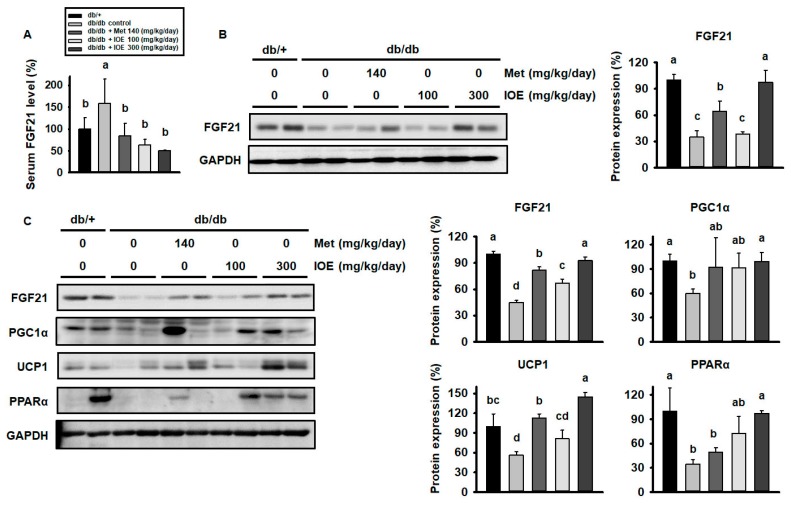
IOE administration increases the expression of FGF21 and browning-associated proteins in WAT. (**A**) The protein level of FGF21 in muscle, (**B**) serum FGF21, and (**C**) expression of FGF21 and mitochondrial proteins in WAT. All data are expressed as mean ± SD (*n* = 6). Values with different letters are significantly different; *p* < 0.05 (a > b > c > d).

To investigate the mechanism by which IOE treatment reduces blood glucose and body mass in *db/db* mice, we next measured the expression of FGF21, an important metabolic regulator, in muscle and WAT, and its serum concentration. As shown in Figure 6A, the serum FGF21 concentration was higher in *db/db* mice than in db/+ mice, but this difference was less pronounced in IOE-treated mice. As shown in Figure 6B, *db/db* mice displayed lower muscle FGF21 protein levels than db/+ mice, but this difference was much smaller in the metformin and IOE-treated groups. The same trend was apparent in WAT (Figure 6C). As shown in Figure 6C, *db/db* mice also demonstrated lower expression of mitochondrial proteins in WAT, including PGC1α, PPARα, and UCP1, than db/+ mice, differences that were also largely abolished in the metformin and IOE-treated groups. These data suggest that IOE ameliorates the downregulation of mitochondrial proteins in the WAT of *db/db* mice, perhaps via the regulation of FGF21 secretion.

## 3. Discussion

In the present study, we showed that IOE treatment has similar effects to metformin to ameliorate insulin resistance, glucose intolerance, and dyslipidemia in mice, probably through activation of the insulin signaling pathway and upregulation of GLUT4 expression. Moreover, we showed that IOE increases the synthesis and secretion of FGF21, a key metabolic regulator of glucose metabolism, body weight, and energy expenditure. We postulate that IOE suppresses weight gain in the form of fat by upregulating PGC1α and PPARα expression, which regulate the expression of pro-thermogenic molecules such as UCP1 in WAT.

Recent studies have demonstrated a beneficial effect of *Ishige okamurae* in metabolic disease [22,26,27]. We previously demonstrated that IOE treatment ameliorates obesity and hepatic steatosis in HFD-induced obese mice [28]. Moreover, *Ishige okamurae* has been reported to prevent hyperglycemia and insulin resistance in *db/db* mice [29]. In addition, it was recently shown that the index component of IOE, Ishophloroglucin A, inhibits the α-glucosidase activity required for glucose absorption [30]. On the basis of these findings, IOE represents a promising therapeutic candidate for the control of blood glucose and body weight, effects that are probably achieved by upregulating metabolism.

The hyperglycemia that characterizes type 2 diabetes is principally the result of insulin resistance in the liver, where gluconeogenesis and glycogen breakdown are upregulated [31]. Chronic, poorly controlled type 2 diabetes is associated with the development of complications, such as retinopathy, neuropathy, and cardiovascular disease. Antidiabetic medications can cause a variety of adverse effects [32,33], and there is now widespread interest in the use of natural products as complementary therapies for diabetes, such that the number of studies conducted regarding the action of functional ingredients has been increasing. Here, we compared the antidiabetic efficacy of IOE and metformin, the first-line treatment for type 2 diabetes, and found that IOE had similar effects to ameliorate hyperglycemia and dyslipidemia in *db/db* mice. Moreover, IOE treatment reduced the concentration of glycated HbA1c, a marker of long-term glycemic status, consistent with similar effects of IOE and metformin to ameliorate insulin resistance and glucose intolerance.

Insulin regulates glucose metabolism in peripheral tissues, principally muscle, liver, and WAT [6]. These effects are mediated through binding to the insulin receptor and activation of the IRS1/PI3K/Akt signaling pathway [34]. *db/db* mice are deficient in leptin receptor functionality, which results in leptin resistance and low levels of IRS1 phosphorylation [35,36]. Consequently, glucose metabolism, cell cycle progression, and protein synthesis are impaired. Here, we showed that IOE administration leads to higher levels of phosphorylation of IRS1, PI3K, and Akt in the muscle and WAT of diabetic mice. Phosphorylation of intermediates in this pathway is required for insulin-stimulated GLUT4 translocation, and adipose-specific GLUT4 disruption causes insulin resistance [11]. These findings suggest that IOE treatment ameliorates insulin resistance by activating the IRS/PI3K/Akt signaling pathway in muscle and WAT, and in tandem with its effect to increase GLUT4 expression, this results in an amelioration of glucose intolerance.

An increase in visceral fat is associated with the development of insulin resistance, which leads to the creation of a vicious circle in which glucose is used for de novo lipogenesis, which causes further visceral fat accumulation. Several studies show that diabetic mice have a higher body and WAT mass [37], but we previously showed that IOE administration reduces body and WAT mass gain by effects on lipid metabolism in HFD-induced obese mice [28]. Consistent with this previous finding, 5 weeks of IOE treatment reduced body mass, fat mass, and lipid droplet size in *db/db* mice. By contrast, several current antidiabetic medications lower blood glucose but increase body weight [38]. 

FGF21 is known to regulate insulin sensitivity and body weight, and circulating serum FGF21 concentration increases as a result of FGF21 resistance in obesity and diabetes [39,40,41]. In the present study, we showed that IOE ameliorates the reduction in serum FGF21 level induced by the *db/db* genotype. FGF21 is regulated in a PI3K/Akt-dependent manner in muscle [42], and overexpression of Akt1 leads to an increase in FGF21 protein expression, suggesting that its secretion from muscle may be physiologically significant [42]. Treatment with FGF21 increases insulin sensitivity in *db/db* mice [43] and upregulates glucose uptake not only in muscle but also in adipocytes [15]. FGF21-treated mice were shown to have higher insulin sensitivity in insulin target tissues, including WAT, skeletal muscle, and liver, in a hyperinsulinemic-euglycemic clamp study [16]. The present data demonstrate that treatment with IOE upregulates FGF21 expression in muscle and WAT. The concurrent upregulation of the insulin signaling pathway may be the result of greater secretion of FGF21 from muscle and WAT, suggesting that the beneficial effects of IOE on the glucose tolerance and insulin sensitivity of *db/db* mice may be FGF21-dependent. However, the high levels of circulating FGF21 may also be the result of feedback resulting from FGF21 resistance in its target tissues.

FGF21 administration increases energy expenditure, lipolysis, and insulin sensitivity while reducing lipogenesis, in HFD-induced obese mice [16]. The effects of FGF21 in adipose include upregulation of PGC1α and UCP1 expression, which are important for mitochondrial biogenesis and energy expenditure in WAT [20], and FGF21 has been shown to directly induce PGC1α expression in 3T3-L1 cells and human adipocytes [18]. Type 2 diabetes and obesity are associated with impaired mitochondrial function in WAT [44,45]. We have shown that IOE increases PGC1α, PPARα, and UCP1 protein expression alongside an increase in FGF21, suggesting that IOE may induce FGF21 synthesis and secretion, resulting in an upregulation of mitochondrial oxidation and energy expenditure. Such effects would explain the lower body mass of IOE-treated *db/db* mice. Induction of FGF21 expression via PPARα plays an important role in the activation of fatty acid oxidation and lipolysis in WAT and liver [46,47]. Therefore, IOE may have an effect on liver glucose metabolism through this mechanism. However, these potential mechanisms must be further evaluated in future studies.

## 4. Materials and Methods 

### 4.1. Materials

Metformin was obtained from Cayman Chemical (Ann Arbor, MI, USA). Anti-phospho (Ser473) Akt (#9271) and total Akt (#9272) antibodies were purchased from Cell Signaling Technology (Danvers, MA, USA). Antibodies against GLUT4 (sc-7938), p-IRS1 (Tyr632) (sc-17196), IRS1 (sc-559), p-PI3K (Tyr508) (sc-12929), PI3K (sc-7174), PGC1α (sc-13067), PPARα (sc-9000), UCP1 (sc-6529), and glyceraldehyde 3-phosphate dehydrogenase (GAPDH) (sc-365062) were purchased from Santa Cruz Biotechnology (Dallas, Texas, USA). FGF21 (ab64857) was purchased from Abcam (Cambridge, UK).

### 4.2. Preparation of IOE

**Figure 7:**
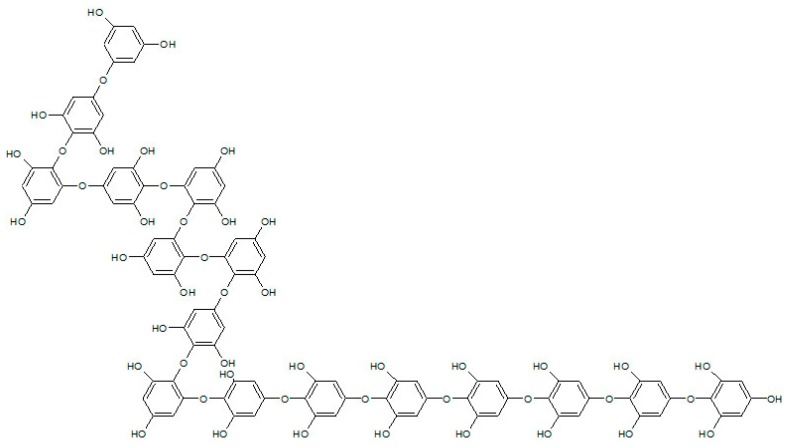
Chemical structure of ishophloroglucin A.

IOE (Lot No. SW8D10SA) was acquired from Jeju National University (Jeju, Korea). *Ishige okamurae* was extracted using 50% ethanol for 24 h at room temperature, then concentrated by vacuum evaporation at 40 °C, filtered, and then subjected to spray drying, and IOE powder was prepared. The total polyphenol content of the IOE was 4.2% [28]. IOE component analysis was performed using a high-performance liquid chromatography (HPLC) system from Agilent Technologies (Santa Clara, CA, USA), an Agilent Poroshell 120 EC-C18 column, and a UV detector (230 nm). The mobile phase was composed of mixtures of solvent A (0.1% formic acid in water) and solvent B (acetonitrile containing 0.1% formic acid). Elution was performed using a 5%–40% B gradient over 40 min, followed by a gradient to 100% B over 10 min, then a 10 min re-equilibration of the column. The flow rate was kept constant at 0.3 mL/min, and the injection volume was 10 μL [30]. According to composition analysis data of Ryu at al., the composition of IOE investigated two predominant peaks, diphlorethohydroxycarmalol (DPHC) and ishophloroglucin A [30]. DPHC (C_24_H_16_O_13_, 512.06 g/mol) was detected at 20.8 min [48]. The index component of IOE, ishophloroglucin A was identified at 35.4 min, and the IOE had an ishophloroglucin A content of 61.5 µg/ml ± 20% (1.24% *w/v* ± 20% of this). It has previously been reported that ishophloroglucin A comprises 29.5% of the total polyphenol content of IOE [28]. As shown in Figure 7, the molecular formula and molecular weight of ishophloroglucin A are C_96_H_66_O_48_ (1986.26 g/mol). Detailed molecular information of IOE such as LC/MS and ^1^H and ^13^C NMR analysis were described in Reference [30].

### 4.3. Animals and Treatments

Five-week-old male *db/db* and lean *db*/+ mice were purchased from CERJ Janvier (Le Genest Saint Isle, France). The animal care and experimental procedures were approved by the Institutional Animal Care and Use Committee (IACUC) of CHA University (IACUC Approval Number 18003). The mice had ad libitum access to water and chow diet, were housed under a 12/12 h light/dark cycle, and had 1 week to acclimate prior to the study. Their food intake was recorded weekly. The mice were randomized to receive the following treatments for 5 weeks: oral administration of vehicle (0 mg/kg), metformin (140 mg/kg), or IOE (100 or 300 mg/kg) (*n* = 8). At the end of the study, the mice had their food withdrawn overnight and were euthanized using CO_2_, and their skeletal muscles, and visceral and subcutaneous fat pads, were excised. The WAT depots and liver were immediately weighed, and all the tissues were snap-frozen and stored at −80 °C.

### 4.4. Oral Glucose and Intraperitoneal Insulin Tolerance Testing 

Five-week-old male ICR mice were purchased from Japan SLC Inc., randomly allocated to four groups (*n* = 6 per group), and fasted overnight, while having free access to water. The mice were then administered orally with 1.5 g/kg glucose, 1.5 g/kg glucose and 100 or 300 mg/kg IOE, or 1.5 g/kg glucose and 140 mg/kg metformin (positive control). Blood glucose was then determined at 0, 30, 60, and 120 min after the administration of glucose.

Intraperitoneal insulin tolerance testing (IPITT) was performed in the morning on mice fasted overnight. Mice were injected intraperitoneally with 0.05 U/kg insulin, and some were also orally administered either 100 or 300 mg/kg IOE. A further negative control group did not receive insulin. Tail vein blood samples were collected at 0 (before the glucose challenge), 30, 60, 90, 120, and 150 min.

### 4.5. Measurement of Blood Glucose, Body Mass, and Water Intake

Fasting glucose was measured in the *db/db* mice and lean *db*/+ mice at the same time weekly in tail vein blood after the removal of food for 12 h. Postprandial blood glucose was also measured in fed *db/db* mice and lean *db*/+ mice. Water intake was measured twice every 1 weeks per cage.

### 4.6. Serum Lipid Profile Analyses

At the end of the 5 weeks treatment period, the *db/db* mice were fasted overnight and a blood sample was collected by cardiac puncture. After clotting, serum was separated by centrifugation at 4 °C, 3500 rpm for 15 min. Commercial kits were used to measure the serum concentrations of insulin, cholesterol, and TG (Roche, Basel, Switzerland). Absorbance was measured using a Cobas 8000 c702 Chemistry Analyzer (Roche). The serum concentrations of glycated hemoglobin (HbA1c) and FGF21 were determined using commercial kits (Mybiosource, San Diego, CA, USA), according to the manufacturer’s instructions.

### 4.7. Histologic Analysis of WAT

After the mice had been euthanized, their WAT depots were rapidly harvested and fixed in 10% formalin solution. After embedding in paraffin, 10 µm sections were cut and stained with hematoxylin and eosin (H and E) to assess their histologic appearance.

### 4.8. Western Blotting

Muscle and WAT samples were homogenized in lysis buffer (Intron biotechnology, Seongnam Korea) supplemented with a protease and phosphatase inhibitor cocktail (Sigma-Aldrich, St. Louis, MO, USA). The protein concentration of each lysate was determined using the Bradford assay (BioRad, Hercules, CA, USA). Twenty micrograms of protein from each sample were separated by SDS-polyacrylamide gel electrophoresis and transferred to PVDF membranes (BioRad), which were incubated with primary antibodies (1:1000) and goat anti-rabbit or mouse secondary antibodies (BioRad, 1: 5,000). Proteins were visualized using enhanced chemiluminescence, and specific bands were quantified using BioRad Imaging Software.

### 4.9. Statistical Analysis

Data are expressed as means ± SD. Statistical Package for Social Sciences version 12.0 (SPSS, Chicago, IL, USA) was used for all analyses. One-way ANOVA was used, followed by the Tukey post-hoc test, as appropriate. The level of significance was set at *p* < 0.05.

## 5. Conclusions

IOE administration ameliorates insulin resistance and hyperglycemia in *db/db* mice, probably through activation of the IRS/PI3K/Akt pathway and an increase in GLUT4 expression in muscle and WAT. Furthermore, the synthesis and secretion of FGF21, a regulator of glucose metabolism and energy expenditure in muscle and WAT, were also higher in IOE-treated mice, which may be responsible for these effects. IOE also increased PGC1α expression, which regulates the expression of thermogenic factors, such as UCP1, in WAT, an effect that may also have been mediated through FGF21.
